# Direct measurement of proximity-induced magnetism at the interface between a topological insulator and a ferromagnet

**DOI:** 10.1038/ncomms12014

**Published:** 2016-06-27

**Authors:** Changmin Lee, Ferhat Katmis, Pablo Jarillo-Herrero, Jagadeesh S. Moodera, Nuh Gedik

**Affiliations:** 1 Department of Physics, Massachusetts Institute of Technology, Cambridge, Massachusetts 02139, USA; 2 Francis Bitter Magnet Laboratory, Massachusetts Institute of Technology, Cambridge, Massachusetts 02139, USA; 3 Plasma Science and Fusion Center, Massachusetts Institute of Technology, Cambridge, Massachusetts 02139, USA

## Abstract

When a topological insulator (TI) is in contact with a ferromagnet, both time-reversal and inversion symmetries are broken at the interface. An energy gap is formed at the TI surface, and its electrons gain a net magnetic moment through short-range exchange interactions. Magnetic TIs can host various exotic quantum phenomena, such as massive Dirac fermions, Majorana fermions, the quantum anomalous Hall effect and chiral edge currents along the domain boundaries. However, selective measurement of induced magnetism at the buried interface has remained a challenge. Using magnetic second-harmonic generation, we directly probe both the in-plane and out-of-plane magnetizations induced at the interface between the ferromagnetic insulator (FMI) EuS and the three-dimensional TI Bi_2_Se_3_. Our findings not only allow characterizing magnetism at the TI–FMI interface but also lay the groundwork for imaging magnetic domains and domain boundaries at the magnetic TI surfaces.

## Abstract (web summary)

Emergent phenomena at the interface between a topological insulator and a ferromanget reflect broken symmetry of topological state. Here, Lee *et al.* report direct measurement of induced magnetism at the Bi_2_Se_3_-EnS interface, paving the way to understand emergent orders in topological material with broken time reversal symmetry.

## Graphical abstract

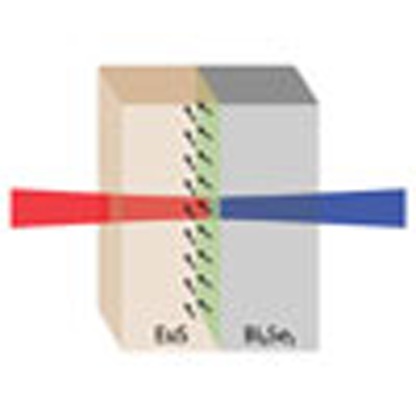

A topological insulator (TI) is a bulk electrical insulator with a two-dimensional metallic surface. The linearly dispersing surface states remain gapless and the spin and momentum channels of the surface electrons are locked to each other as long as time-reversal symmetry (TRS) is preserved^1^,^2^. When TRS is externally broken with moderate perturbations, however, a TI does not simply transform into a trivial insulator. While an energy gap opens up and the spin degeneracy is lifted at the Dirac point^3^, the surface electrons acquire a non-trivial spin texture distinct from that of a TI or a trivial insulator^4^. TRS can be broken on a TI surface either by introducing magnetic dopants or by directly growing a ferromagnetic insulator (FMI) on top. While most of the past research has been focused on the former approach^3^,^4^,^5^,^6^,^7^,^8^, the TI–FMI interfaces provide a cleaner method by inducing a more uniform long-range magnetic order across the TI surface. Moreover, such interfaces do not introduce impurity scattering centres to the surface electrons.

Recently, the EuS–Bi_2_Se_3_ interface has emerged as a novel TRS-broken TI system^9^,^10^. By itself, EuS is an electrical insulator (energy gap=1.65 eV) that becomes ferromagnetic below ∼17 K (=*T*_Curie_), and its spins are aligned parallel to the basal plane^11^. However, when EuS is in close proximity to Bi_2_Se_3_, electrons at the EuS–Bi_2_Se_3_ interface acquire a canted magnetic moment, causing TRS to be broken at the TI surface. So far, conventional magnetometry and linear magneto-optical Kerr effects have been used to measure the magnetic properties of this system^9^,^10^, but these measurements were dominated by the contribution from the bulk magnetism of the EuS film. Furthermore, the nature of proximity-induced magnetism at the TI–FMI interface is not completely understood, and the lack of experimental probes limits access to such buried regions.

Magnetic second-harmonic generation (MSHG) is a powerful optical tool capable of selectively probing interface magnetism^12^, as symmetry arguments restrict the MSHG signal to be generated only from magnetized surfaces and interfaces of a centrosymmetric material. MSHG has been successfully applied to measure magnetism either at the Fe surface^13^, or at the Co/Cu (ref. 14) and the SrTiO_3_/La_1−*x*_Sr_*x*_MnO_3_ interfaces^15^ to name a few.

In this work, we reveal both the magnetic and crystal symmetries at the interface of EuS–Bi_2_Se_3_ heterostructures with MSHG. We simultaneously measure the in-plane and out-of-plane components of the proximity-induced ferromagnetism at the buried EuS–Bi_2_Se_3_ interface.

## Results

### Experimental procedure

**Figure 1:**
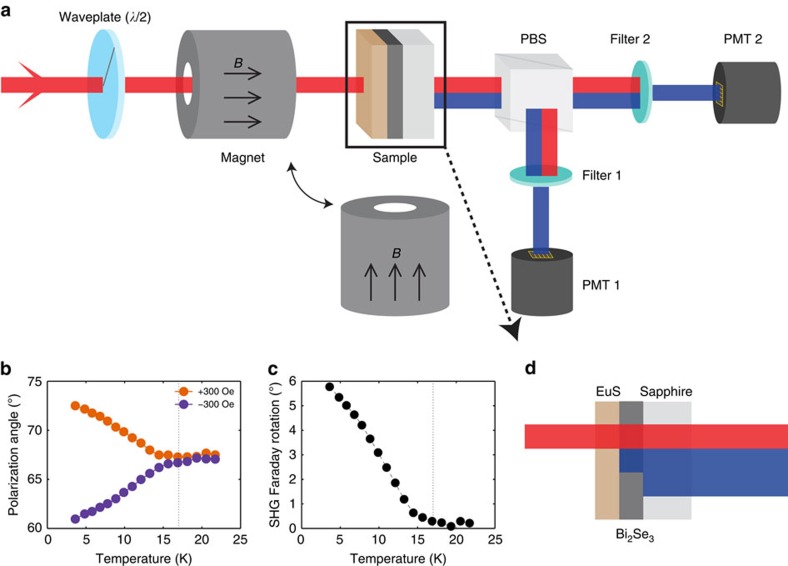
SHG Faraday rotation. (**a**) Experimental set-up. PBS, polarizing beam splitter; PMT, photomultiplier tube. Rotation of the SHG polarization plane was calculated by using two PMTs that separately measure SHG intensities of *s* and *p* polarizations, respectively. Either an in-plane or an out-of-plane magnetic field was applied to the sample. (**b**) Output SHG polarization angle and (**c**) SHG Faraday rotation angle plotted against temperature for the 7 nm (EuS)–7 QL (Bi_2_Se_3_) sample. (**d**) In EuS–Bi_2_Se_3_ heterostructures, surface SHG is generated from the EuS–Bi_2_Se_3_ and Bi_2_Se_3_–sapphire interfaces. In **a** and **d** red and blue lines correspond to the laser beams of *ω* and 2*ω* frequencies.

**Figure 2:**
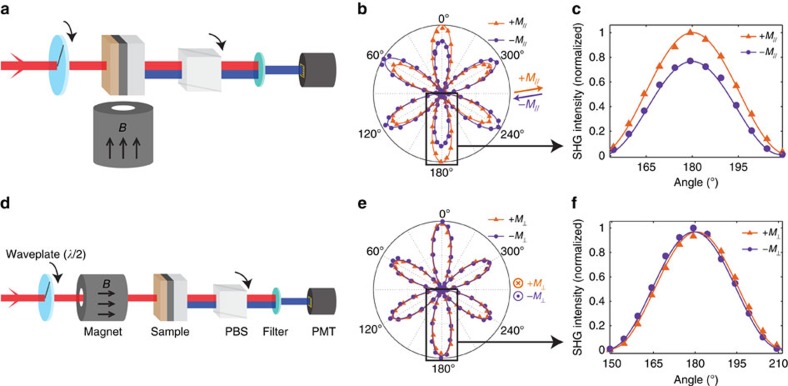
MSHG-RA patterns of the EuS–Bi_2_Se_3_ heterostructures. (**a**,**d**) Experimental set-up of the MSHG-RA measurements. Magnitude of SHG was measured as a function of input polarization angle while an (**a**) in-plane or an (**d**) out-of-plane magnetic field was applied to the sample. A set of a half-wave plate and a polarizer was rotated simultaneously so that the output SHG polarization was set to be either parallel or perpendicular to the input polarization. Only the parallel polarization measurements are shown here. (**b**) SHG Intensity as a function of input polarization angle from a 7 nm (EuS)–7 QL (Bi_2_Se_3_) sample. In-plane magnetic fields of +300 (orange) and −300 Oe (purple) were applied to the sample at 4 K. (**c**) An enlarged plot of the rectangular area in **b**. (**e**) The same plot as **b** but out-of-plane magnetic fields of +4,000 (orange) and −4000 Oe (purple) were applied to the sample. A small rotation of the MSHG-RA pattern is observed in **f**, which is an enlarged plot of the rectangular area in **e**. In **b** and **e** magnetic field directions are denoted by the orange and purple arrows, dots and crosses.

The MSHG experiment was carried out in a transmission geometry (Fig. 1a). A magnet was used to produce either an in-plane (up to 300 Oe) or an out-of-plane magnetic field (up to 4,000 Oe) at the sample, and a set of a half-wave plate and a polarizer was placed to control the input and output light polarizations, respectively. The experiment was performed under two different configurations—two detectors (photomultiplier tubes) were simultaneously used to measure the second-harmonic generation (SHG) Faraday rotation (Fig. 1a) or a single detector was used to measure the SHG intensity as a function of input polarization angle ((MSHG-RA: MSHG rotational anisotropy) patterns^16^ shown in Fig. 2b,e). A detailed description of the optical measurements can be found in Supplementary Note 1.

### SHG Faraday rotation

Under the first experimental configuration (Fig. 1a), SHG Faraday rotation angles were measured from a 7 nm–7 QL (EuS and Bi_2_Se_3_ thicknesses, respectively; QL: quintuple layer) hybrid heterostructure sample. When a magnetic field (300 Oe) is applied to the sample along the in-plane direction, magnetization at the interface causes the SHG polarization plane to be rotated. When the direction of the magnetic field is reversed, the polarization plane is rotated in the opposite direction. The amount of this polarization rotation is defined as the SHG Faraday rotation. In Fig. 1b,c, it is clearly seen that a large SHG Faraday rotation sets in slightly below 17 K (*T*_Curie_), verifying its magnetic origin. It is also important to note that SHG Faraday rotation is allowed in samples with an in-plane magnetization at normal incidence, unlike the linear Faraday effect that is only sensitive to out-of-plane magnetization^12^.

Since there are four distinct interfaces in a EuS–Bi_2_Se_3_ heterostructure film (Fig. 1d), it is important to identify the interfaces contributing to SHG. We thus prepared both a bare sapphire (0001) substrate and a EuS film (5-nm thick) grown on sapphire (0001) as control samples. We could not detect any measurable SHG signal from either sample down to 4 K. The same observation holds even when a magnetic field was applied either in the in-plane (up to 300 Oe) or the out-of-plane (up to 4,000 Oe) direction. Therefore, we conclude that all surface dipole SHG contributions of a EuS–Bi_2_Se_3_ heterostructure arise only from the EuS–Bi_2_Se_3_ and Bi_2_Se_3_–sapphire interfaces, and not from the top EuS surface or the bottom sapphire surface (Fig. 1d). We also note that since SHG is not generated from the bulk of the magnetized EuS film, SHG Faraday rotation arises from the proximity-induced ferromagnetism at the top surface of the Bi_2_Se_3_ film.

### In-plane magnetism

To quantitatively characterize the strength of interface ferromagnetism and reveal the magnetic symmetry of EuS–Bi_2_Se_3_ heterostructures, we took MSHG-RA measurements from the same (7 nm–7 QL) heterostructure sample. SHG intensity was measured as a function of input polarization angle, while an in-plane (Fig. 2a) or an out-of-plane (Fig. 2d) magnetic field was applied to the sample. The output polarization of SHG was selected to be either parallel (PA) or perpendicular (CR, or crossed) to the input polarization. At 295K, no discernible change in the RA pattern was measured within the resolution of the technique in the presence of a magnetic field. However, at 4 K (<*T*_Curie_), a large difference in the MSHG-RA pattern (PA polarization set-up) was observed between +300 and −300 Oe of in-plane magnetic fields applied to the sample (Fig. 2b,c). Data from the CR polarization set-up is shown in Supplementary Figs 1 and 2.

The MSHG-RA patterns can be divided into contributions from the non-magnetic crystal and the interface ferromagnetism^12^,^17^:


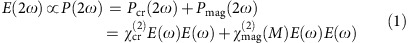


where *P*(2*ω*) is the second-order electric polarization, *χ*^(2)^ is the second-order electric susceptibility tensor, *M* is the interface magnetization and *E*(*ω*) is the electric field of the incident light of frequency *ω*. The tensor 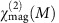 obtains a non-zero value only below *T*_Curie_. The MSHG-RA signal consists of the following three terms^18^ (a detailed description of the theoretical model and the fitting procedure is provided in Supplementary Note 2):


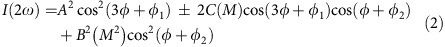


The first SHG term containing *A*^2^ purely originates from the crystal, and the third term containing *B*^2^(*M*^2^) is from the interface magnetism. The second term is caused by the interference of the crystalline and magnetic SHG terms. The interference term *C*(*M*) is odd with respect to the magnetization of the sample, and thus gives rise to the large nonlinear Faraday rotation in Fig. 1b,c and the differential MSHG-RA patterns in Fig. 2b,c. The MSHG-RA data due to in-plane magnetization are fitted remarkably well to equation (2), as shown by the orange and purple dots (data) and lines (fits) in Fig. 2b,c.

### Out-of-plane magnetism

We now turn to the effect of out-of-plane magnetization on the MSHG-RA patterns. Out-of-plane magnetic moments are more important as they are responsible for breaking TRS in a TI system. While an in-plane magnetization is easily induced even with a few tens of Oersteds of magnetic field, an out-of-plane magnetization requires a stronger magnetic field (up to 3 T for complete saturation)^9^. Since it is difficult to align the out-of-plane magnetic domains without affecting the in-plane domains, we applied a tilted (∼4°) magnetic field to the sample so that both the in-plane and the out-of-plane magnetizations are induced along a preferred direction at the interface. As described in ref. 17, an out-of-plane magnetization causes the MSHG-RA patterns to be rotated:


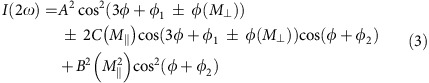


where *M*_||_ and *M*_⊥_ are the in-plane and out-of-plane magnetizations, respectively, and *φ*(*M*_⊥_) is the rotation angle of the RA pattern due to an out-of-plane magnetization. Figure 2e,f shows the MSHG-RA patterns when a tilted out-of-plane magnetic field of 4000 Oe is applied towards (orange) or away from (purple) the sample. A small rotation of the MSHG-RA pattern was observed, indicating the presence of a net out-of-plane magnetic moment at the EuS–Bi_2_Se_3_ interface. We note that the magnitude of polarization rotation angle (∼1°) measured in this MSHG-RA pattern is orders of magnitude larger than that reported from a previous linear magneto-optic Kerr effect measurement (∼100 μrad)^10^.

### EuS thickness dependence

**Figure 3:**
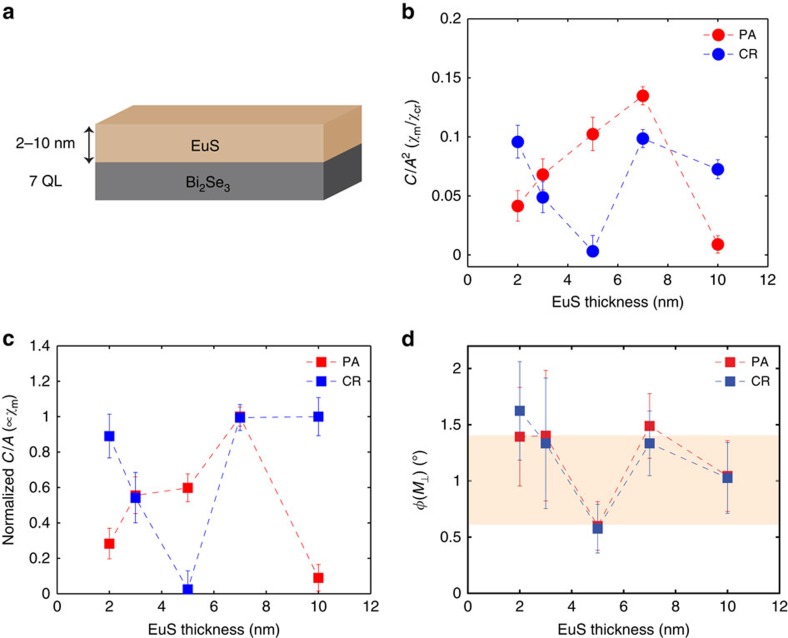
EuS thickness dependence. (**a**) MSHG parameters were measured from samples of various EuS thicknesses (2–10 nm). Bi_2_Se_3_ thickness is fixed at 7 QL. (**b**) *C*(*M*)/*A*^2^ and (**c**) *C*(*M*)/*A* are plotted as a function of EuS thickness. Both values exhibit a strong variance over different EuS thicknesses. (**d**) Out-of-plane response *φ*(*M*_⊥_) for different EuS thicknesses. In **b**–**d** error bars represent the uncertainties of least square fitting of data to the theoretical model.

To further verify that the MSHG signal comes from the EuS–Bi_2_Se_3_ interface, and not from the magnetic bulk of the EuS film, we took MSHG-RA measurements from samples of different EuS thicknesses. While the Bi_2_Se_3_ thickness was fixed at 7 QL, the EuS thickness was varied from 2 to 10 nm (Fig. 3a). In Fig. 3b,c, values of *C*(*M*)/*A*^2^ and *C*(*M*)/*A* (normalized) are plotted as a function of EuS thickness. Since 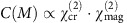 and 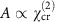, it follows that 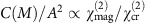 and 
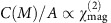
. For both quantities, two sets of data can be acquired for the PA and CR polarization set-ups. If the magnetic signal was to come from the bulk of EuS film, one would expect a monotonically increasing behaviour of *C*(*M*)/*A*^2^ and *C*(*M*)/*A* as a function of EuS thickness, contrary to what is observed in Fig. 3b,c. A striking feature here is the large variation of the magnetic signal across different EuS thicknesses.

There are two possible explanations for the fluctuations in the magnetic signal. First, since MSHG is interface sensitive, *C*(*M*)/*A*^2^ and *C*(*M*)/*A* values may simply correspond to the different interface quality of each sample. In this case, however, the magnetic signal for the PA and CR polarization set-ups should increase or decrease together, unlike Fig. 3b,c where the PA and CR magnetic signals often follow an opposite trend with respect to each other. Another possibility is the existence of spin-polarized quantum well states in the EuS–Bi_2_Se_3_ heterostructures similar to those observed in the Co/Cu films^14^ and Au/Co/Au trilayers^19^. When the thickness of a material becomes finite, its electrons can form a quantum well state bound inside the film (Supplementary Fig. 3). Moreover, in a ferromagnetic system, the majority and the minority spin states can become relatively more or less bound depending on the strength of the potential barrier exerted by the neighbouring material. Such spin-polarized quantum well states can give rise to an oscillating behaviour of the magnetic signal as a function of sample thickness^14^,^19^ (see Supplementary Note 3 for details). A detailed band structure calculations of the EuS–Bi_2_Se_3_ heterostructures can help to answer this question^20^.

The out-of-plane magnetic signal is more uniform across samples of different EuS thicknesses (Fig. 3d), compared with the in-plane response. It is important to note that all five samples exhibit both in-plane and out-of-plane magnetizations, and that the magnetic signal does not uniformly increase with EuS thickness, indicating the strong interface sensitivity of MSHG.

### Bi_2_Se_3_ thickness dependence

**Figure 4:**
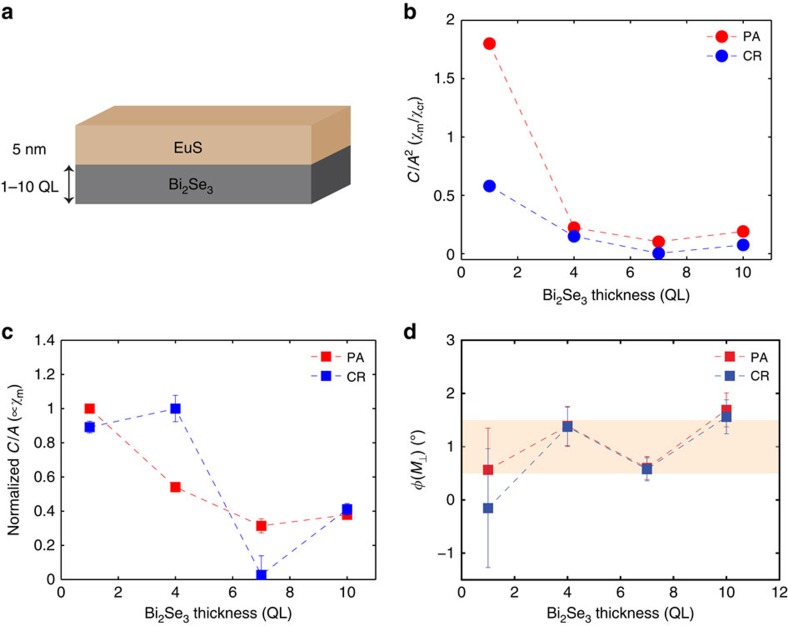
Bi_2_Se_3_ thickness dependence. (**a**) MSHG parameters were measured from samples of various Bi_2_Se_3_ thicknesses (1–10 QL). EuS thickness is fixed at 5 nm. Both (**b**) *C*(*M*)/*A*^2^ and (**c**) *C*(*M*)/*A* show a decreasing behaviour, possibly due to the increased bulk contributions and SHG absorption from thicker Bi_2_Se_3_ films. (**d**) Out-of-plane response *φ*(*M*_⊥_) for different Bi_2_Se_3_ thicknesses. In **b**–**d** error bars represent the uncertainties of least square fitting of data to the theoretical model.

To rule out the possibility that MSHG probes bulk magnetism of Bi_2_Se_3_ films, we now proceed to measurements taken on samples of different Bi_2_Se_3_ thicknesses. While EuS thickness was fixed at 5 nm, Bi_2_Se_3_ thickness was varied from 1 to 10 QL (Fig. 4a). As shown in Fig. 4b, 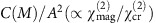 sharply decreases with Bi_2_Se_3_ thickness, which is due to the increasing non-magnetic bulk SHG contributions from thicker samples. It has been reported from earlier studies on Bi_2_Se_3_ single crystals that SHG contains bulk contributions^21^,^22^ due to a band-bending effect near the surface. A similar band-bending effect in Bi_2_Se_3_ films induced by the SiC substrate was also reported from a previous angle-resolved photoemission (ARPES) measurement^23^. When 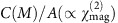 is plotted against Bi_2_Se_3_ thickness (Fig. 4c), we still observe a decreasing trend. Such behaviour is attributed to the increasing absorption of MSHG from thicker Bi_2_Se_3_ samples (penetration depth for a 400 nm laser beam is ∼10 nm, or 10 QL (ref. 24)), and indicates that MSHG is not generated from the bulk of the Bi_2_Se_3_ film. Out-of-plane response is more uniform across samples of different Bi_2_Se_3_ thicknesses (Fig. 4d), except for the 1-QL sample that barely exhibits a non-zero value.

## Discussion

We have measured both the in-plane and out-of-plane components of ferromagnetism induced at the EuS–Bi_2_Se_3_ interface due to the proximity effect, and demonstrated the interface sensitivity of MSHG. The ability to simultaneously probe interface magnetizations along both directions provides a completely unique pathway towards studying the surfaces of magnetic TI systems. One particular interest in the field of magnetic TIs is the predicted existence of chiral edge states along the ferromagnetic domain boundaries^1^,^2^. Such dissipationless chiral currents can be potentially used for realizing topological magnetoelectric effects and novel devices based on spintronics. Our MSHG capabilities can be readily extended to imaging magnetic domains and domain boundaries at the interface. The exclusive feature of MSHG imaging absent in other scanning probe or optical techniques is the ability to distinguish the magnetic domains aligned along the in-plane or the out-of-plane directions^25^. It can be a powerful tool for identifying magnetic domain walls (Bloch or Néel)^12^ or visualizing spin texture at the domain boundary of various magnetic TI systems.

Another approach is to combine the current MSHG set-up with a pump–probe spectroscopy to investigate the non-equilibrium dynamics of interface magnetism, which is a largely unexplored field in the study of magnetic TIs^22^,^26^. Measuring the relaxation dynamics of spin, electronic and lattice degrees of freedom, and studying how these parameters are coupled to one another should provide more insight into the mechanism of proximity-induced ferromagnetism.

The capabilities of reducing band-bending effects and tuning chemical potential near the Dirac point (for example, through electrostatic gating)^7^,^8^,^27^ should further enhance the effectiveness of MSHG as a probe of the buried interface that is not easily accessed by other conventional techniques.

## Methods

### Sample growth

The epitaxial growth of EuS–Bi_2_Se_3_ bilayer was performed in a custom-built molecular beam epitaxy (MBE) system, under a base pressure of 2 × 10^−10^ Torr, equipped with effusion cells for Bi and Se thermal evaporation, and an electron beam evaporator for EuS deposition. The interface formation was between EuS and Bi_2_Se_3_ was monitored by an *in situ* reflection high-energy electron diffraction unit. Double-side-polished sapphire (0001) epi-ready substrate was prepared *in situ* with several baking treatments to create atomically flat surfaces, which were ensured by reflection high-energy electron diffraction measurements (see Supplementary Note 4 and Supplementary Figs 4 and 5 for details). On a well-prepared surface, Bi and Se were co-evaporated with a 1:15 flux ratio to the substrate at a defined temperature (240±5 °C). Owing to the stable 2:3 phase of Bi–Se compounds, self-adjusting takes place at the final composition (Bi_2_Se_3_) when a 1:15 flux ratio is used at the growth temperature. To avoid kinetic surface roughening, a 1–2-Å min^−1^ growth rate was used to produce a smooth surface. Owing to the high reactivity of Eu atoms and dissociation problems of sulfur, EuS was evaporated congruently from a single electron-beam source onto the Bi_2_Se_3_ layer at a rate of 0.5–0.6 Å s^−1^ under ultra-high vacuum (UHV) condition. As a final step, all bilayer (EuS–Bi_2_Se_3_) films were covered *in situ* with ∼5 nm amorphous Al_2_O_3_ at room temperature as a protection layer in the same deposition chamber.

### Optical measurements

All MSHG measurements were carried out using a Ti:sapphire laser amplifier system operating at 785 nm, with a pulse duration of 50 fs. In all measurements, the incident laser pulse was focused onto a 50-μm spot at the sample using a standard convex lens, and the fluence of the incident laser excitation did not exceed 3 mJ cm^−2^, which is below the damage threshold of all heterostructure samples. A ring-shaped neodymium magnet was used to produce a magnetic field at the sample, and fluorescent filters were placed before the detectors to prevent any residual fundamental frequency laser pulses from being detected.

### Data availability

The data that support the findings of this study are available from the corresponding author on request.

## Additional information

**How to cite this article:** Lee, C. *et al.* Direct measurement of proximity-induced magnetism at the interface between a topological insulator and a ferromagnet. *Nat. Commun.* 7:12014 doi: 10.1038/ncomms12014 (2016).

## Supplementary Material

Supplementary Information Supplementary Figures 1-5, Supplementary Notes 1-4 and Supplementary References
