# Supplementary material for: Direct measurement of proximity-induced magnetism at the interface between a topological insulator and a ferromagnet
Source: Nat Commun. 2016 Jun 27;7:12014. doi: 10.1038/ncomms12014 (PMC4931222; doi:10.1038/ncomms12014)
Supplement: Supplementary Information — Supplementary Figures 1-5, Supplementary Notes 1-4 and Supplementary References [file ncomms12014-s1.pdf]

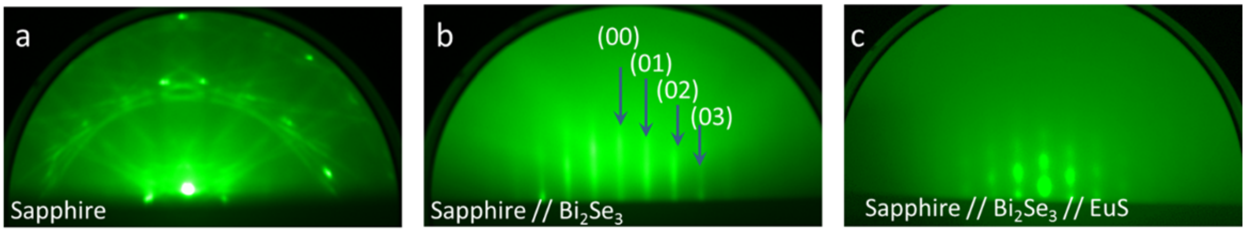


**Supplementary Figure 1 | In situ RHEED patterns during the growth of a EuS-$Bi_2Se_3$ bilayer film.** RHEED patterns of **a,** a heat treated sapphire surface, **b,** a 7 QL $Bi_2Se_3$ film and **c,** a 5 nm-7QL EuS-$Bi_2Se_3$ bilayer heterostructure.

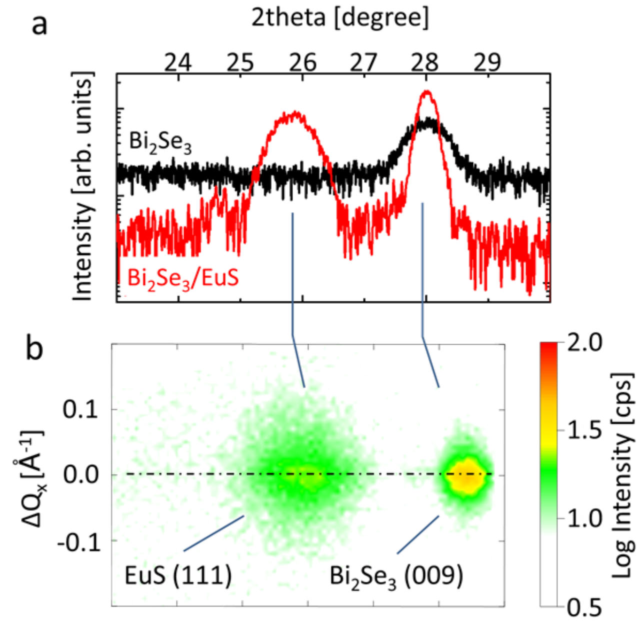


**Supplementary Figure 2 | Ex situ XRD patterns from a 5 nm-7 QL (EuS-$Bi_2Se_3$) bilayer film. a,** XRD line scan along the EuS(111) and $Bi_2Se_3$(009) Bragg peaks, or the line cut (dashed line) of the reciprocal space map in **b**. For comparison, a control $Bi_2Se_3$ film without EuS is also shown in the same figure (black line scan), in which the EuS(111) peak is missing. **b,** Reciprocal space map at the vicinity of the EuS(111) and $Bi_2Se_3$(009) Bragg peaks, where $Q_{\mathrm{x}}$ is defined as $(2\pi/\lambda)[\cos(2\theta-\omega)-\cos(\omega)]$.

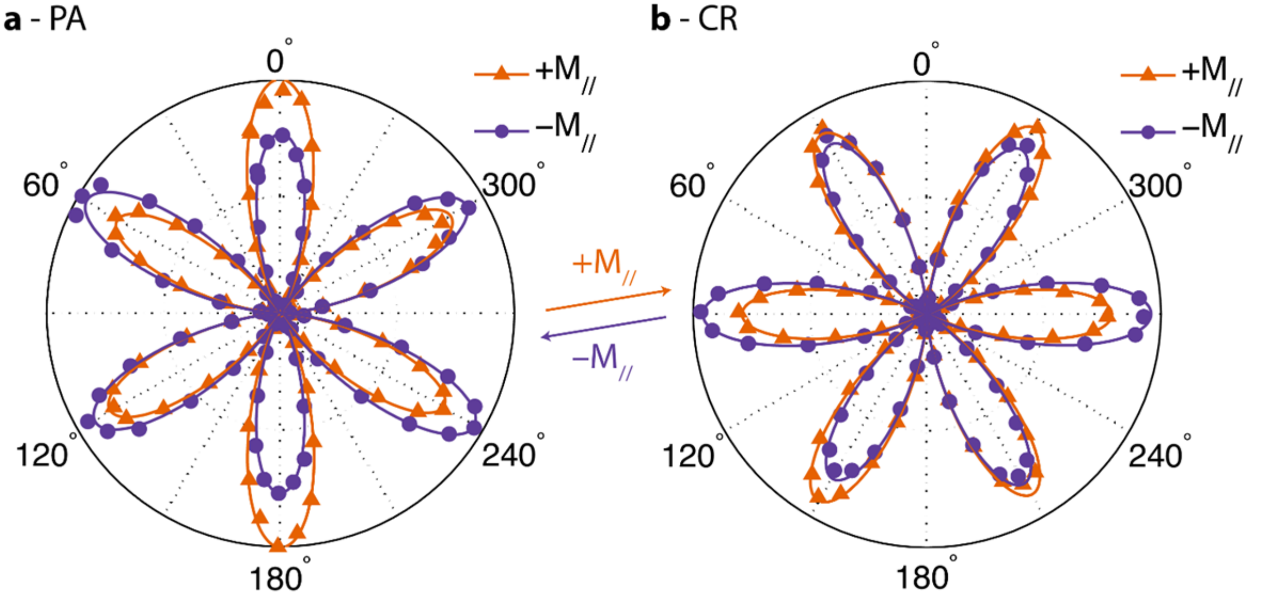


**Supplementary Figure 3 | MSHG-RA patterns of a 7 nm-7 QL heterostructure film under in-plane magnetic fields.** MSHG-RA patterns are shown for the **a,** PA and **b,** CR polarization setups. Magnetic field directions are denoted by the orange and purple arrows.

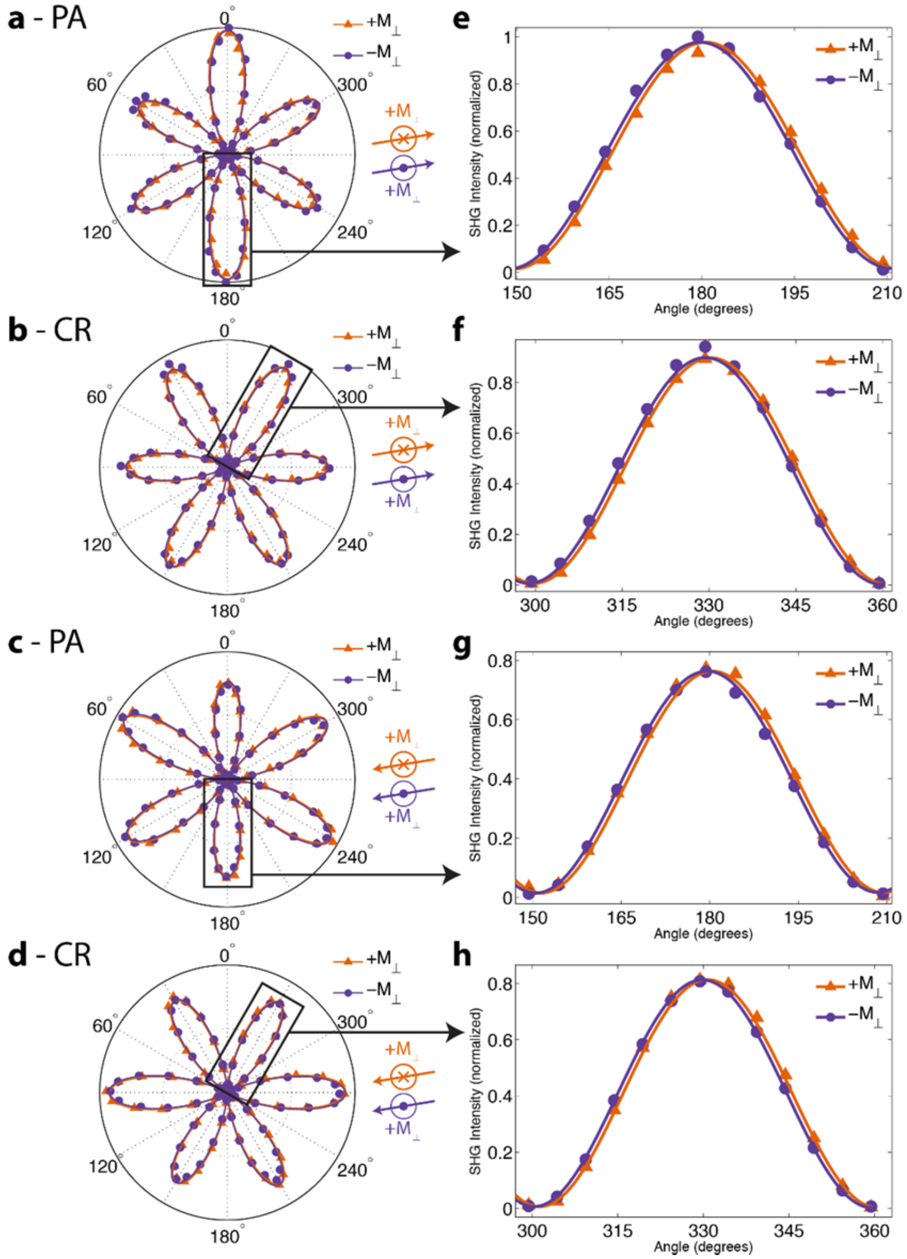


**Supplementary Figure 4 | MSHG-RA patterns of a 7 nm-7 QL heterostructure film under out-of-plane magnetic fields. a-d,** MSHG-RA patterns are shown for the PA (**a,c**) and CR (**b,d**) polarization setups while a canted magnetic field (∼4°) was applied to the sample. Canted magnetization directions are denoted by arrows, circles, and crosses next to each figure. **e-h,** enlarged plots of the rectangular area in **a-d**.

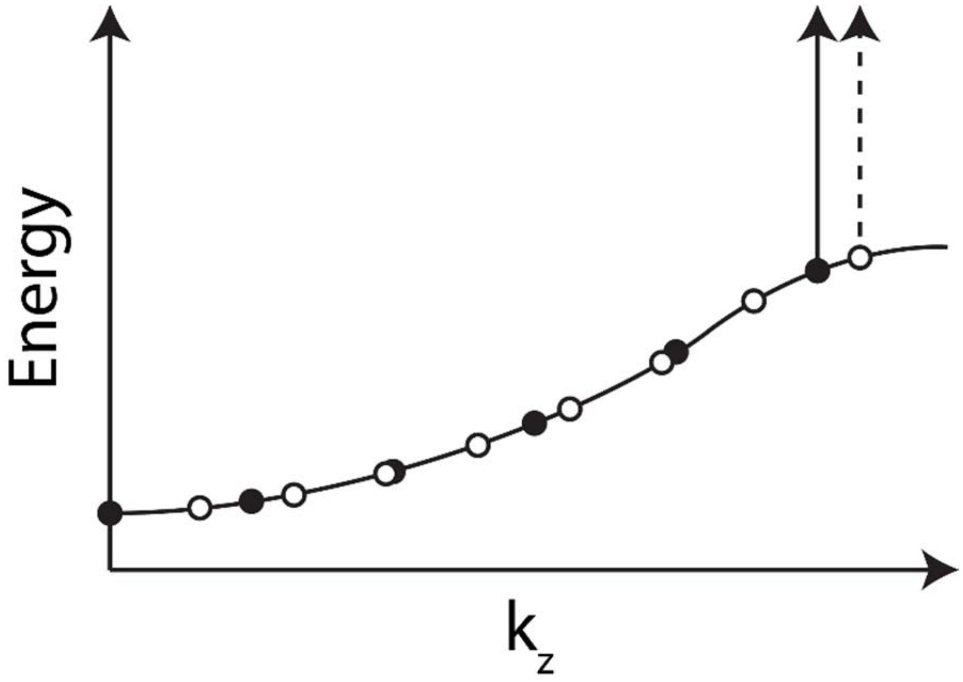


**Supplementary Figure 5 | Band structure of a model QWS**. In two QWSs of different thicknesses, optical transitions take place at different points of the Brillouin zone. Filled and empty circles correspond to the $k_z$ points of the thicker and the thinner QWS, respectively.

## Supplementary Note 1: Detailed sample growth and structural analysis

### (1) Interface formation

The growth process and the morphological evaluation of the individual surfaces were tracked with an in situ reflection high-energy electron diffraction (RHEED) apparatus, in which the electrons probe the top-most layer at a grazing angle. Supplementary Fig. 1a shows the RHEED snapshot pattern of the sapphire substrate after a careful surface preparation, and the final surface RHEED image of a 7 QL $Bi_2Se_3$ is shown in Supplementary Fig. 1b. After the growth of $Bi_2Se_3$, the substrate temperature was reduced down to ~60-70 ℃ for the growth of EuS. While growing EuS on $Bi_2Se_3$, the two dimensional streaky RHEED pattern indicating a smooth $Bi_2Se_3$ surface (Supplementary Fig. 1b) transforms into a three dimensional spotty pattern, which corresponds to a truncated flat island due to a lack of proper mobility at the EuS surface (Supplementary Fig. 1c). Surface roughness of ~0.15 nm, ~0.84 nm, and ~1.2 nm were confirmed with ex situ X-ray reflectivity measurements, respectively, for the sapphire substrate and the $Bi_2Se_3$ and EuS films.

### (2) Structural characterization

In order to obtain a detailed information about the crystal structure and the surface and interface correlations, the films were investigated by X-ray diffraction (XRD) performed ex-situ with Cu-$K_\alpha$ $(\lambda = 1.54\ \text{Å})$. In order to verify the structural relation between the grown layers, XRD measurements were recorded at the vicinity of Bragg reflections of bilayers. The high resolution XRD for different bi-layer configurations and pure $Bi_2Se_3$ films are compared with

scans along the growth direction (Supplementary Fig. 2). The profile shows a clear EuS (111) orientation on $Bi_2Se_3$ (009) layers, which indicates a EuS(111)-$Bi_2Se_3$(0001)-$Al_2O_3$(0001) stacking relation of the heterostructure.

## Supplementary Note 2: Detailed optical measurements

All measurements were carried out while the thin film heterostructure samples were kept under high vacuum ($< 5 \times 10^{-6}$ torr) in an optical cryostat. Incoming laser pulses are focused onto a 50 μm spot using a standard convex lens ($f = 20\text{cm}$), and the transmitted laser pulses from the sample are collimated using another convex lens ($f = 12.5\text{cm}$).

**(1) SHG Faraday rotation measurements**

The collimated pulses were then split into two beams of orthogonal polarizations (*s* and *p*) using a Rochon polarizer. Two photomultiplier tubes (PMTs, Hamamatsu H10721-110) were used to separately detect the SHG intensity of the two polarizations, and a fluorescence filter (Thorlabs MF390-18) was placed before each PMT so that the fundamental wavelength (785 nm) laser pulses are spectrally filtered. The output current channel from each PMT was terminated with a 10 kΩ resistor, and the voltage across this resistor was then measured using a standard lock-in technique synchronised with the repetition rate of the amplified laser system (30 kHz).

The polarization angle $\phi$ of the output SHG is calculated as follows:

$$\phi = \frac{E_p(2\omega)}{E_s(2\omega)} = \sqrt{\frac{I_p(2\omega)}{I_s(2\omega)}} \quad (1)$$

where $E_s(2\omega)$, $E_p(2\omega)$, $I_s(2\omega)$ and $I_p(2\omega)$ are the SHG electric fields and intensities of *s* and *p* polarizations, respectively. Therefore, the magnitude of the SHG Faraday rotation angle is

$$\begin{aligned} \Delta\phi &= \phi(+M) - \phi(-M) \\ &= \sqrt{\frac{I_p(2\omega, +M)}{I_s(2\omega, +M)}} - \sqrt{\frac{I_p(2\omega, -M)}{I_s(2\omega, -M)}} \end{aligned} \quad (2)$$

where $\pm M$ refer to magnetisations of opposite signs.

**(2) MSHG-RA measurements**

In MSHG-RA measurements, a Glan-Taylor polarizer was placed after the collimating beam as an analyzer. A half-wave plate and a polarizer were each mounted on a separate computer-controlled rotation stage (Newport PR50CC), and were simultaneously rotated so that the input and output polarizations are either parallel (PA) or perpendicular (CR, or crossed) to each other. A single PMT was used to take MSHG measurements at each input polarization angle.

## Supplementary Note 3: Fitting Procedures of the MSHG-RA data

### (1) In-plane magnetization

When an in-plane magnetic field is applied to the sample, $E(2\omega)$ can be written as a function of the input polarization angle $\phi$, thin film orientation angle $\phi_1$, and the direction of the magnetic field $\phi_2$:[1-4]

$$E(2\omega) = E_{\mathrm{cr}}(2\omega) + E_{\mathrm{mag}}(2\omega)$$

$$= A\cos(3\phi + \phi_1) \pm B(M)\cos(\phi + \phi_2), \quad (3)$$

where the $A\cos(3\phi + \phi_1)$ term corresponds to the $3m$ symmetry of the $Bi_2Se_3$ surface, and the $B(M)\cos(\phi + \phi_2)$ term reflects the strength and direction of the in-plane magnetisation at the EuS-$Bi_2Se_3$ interface. The latter term is odd (changes sign) with respect to magnetisation. Since MSHG intensity, not the electric field strength, was measured in the actual experiment, the MSHG-RA signal consists of the following three terms:

$$I(2\omega) = A^2\cos^2(3\phi + \phi_1)$$

$$\pm 2C(M)\cos(3\phi + \phi_1)\cos(\phi + \phi_2)$$

$$+B^2(M^2)\cos^2(\phi + \phi_2), \quad (4)$$

which in turn can be rewritten as:

$$I(2\omega) = \frac{A^2}{2}\cos(6\phi + 2\phi_1)$$

$$\pm 2C_{\mathrm{PA}}(M)\cos(3\phi + \phi_1)\cos(\phi + \phi_2)$$

$$+\frac{B_{\mathrm{PA}}^2}{2}(M^2)\cos(2\phi + 2\phi_2) + D_{\mathrm{PA}} \quad (5)$$

when the input and output polarizations are parallel (PA) to each other. Similarly, when the input and output polarizations are perpendicular to each other (CR), the MSHG-RA pattern can be described as:

$$I(2\omega) = \frac{A^2}{2}\sin(6\phi + 2\phi_1)$$
$$\pm 2C_{\mathrm{CR}}(M)\sin(3\phi + \phi_1)\sin(\phi + \phi_2)$$
$$+\frac{B_{\mathrm{CR}}^2}{2}(M^2)\sin(2\phi + 2\phi_2) + D_{\mathrm{CR}}. \quad (6)$$

All MSHG-RA data under in-plane magnetic fields were fitted using Supplementary eqs. (5) and (6), as they minimize the number of products of sinusoidal functions. Four sets of data (two magnetization directions and two polarization setups) were fitted to two equations sharing nine parameters ($A$, $B_{\mathrm{PA}}$, $B_{\mathrm{CR}}$, $C_{\mathrm{PA}}$, $C_{\mathrm{CR}}$, $D_{\mathrm{PA}}$, $D_{\mathrm{CR}}$, $\phi_1$, $\phi_2$). In all measurements, fitted values of $B$ were too small compared to the size of their error bars (~1-2% of $A$) to extract any information about the strength of the magnetic signal, and are not shown in this work. Data (squares and circles) and fits (lines) from the 7 nm (EuS)-7 QL ($Bi_2Se_3$) heterostructure sample are shown in Supplementary Fig. 3.

**(2) Out-of-plane magnetization**

Similarly, MSHG data under an out-of-plane magnetic field were fitted using the following two equations, depending on the polarization combinations (PA and CR),

$$\text{PA: } I(2\omega) = \frac{A^2}{2}\cos\big(6\phi + 2\phi_1 \pm \phi_{\mathrm{PA}}(M_\perp)\big)$$

$$\pm 2C_{\mathrm{PA}}(M)\cos\big(3\phi + \phi_1 \pm \phi_{\mathrm{PA}}(M_\perp)\big)\cos(\phi + \phi_2) \tag{7}$$

$$+\frac{B_{\mathrm{PA}}^2}{2}(M^2)\cos(2\phi + 2\phi_2) + D_{\mathrm{PA}}$$

$$\text{CR: } I(2\omega) = \frac{A^2}{2}\cos\big(6\phi + 2\phi_1 \pm \phi_{\mathrm{CR}}(M_\perp)\big)$$

$$\pm 2C_{\mathrm{PA}}(M)\cos\big(3\phi + \phi_1 \pm \phi_{\mathrm{CR}}(M_\perp)\big)\cos(\phi + \phi_2) \tag{8}$$

$$+\frac{B_{\mathrm{CR}}^2}{2}(M^2)\cos(2\phi + 2\phi_2) + D_{\mathrm{CR}}.$$

As described in the main text, a tilted ($\sim 4°$) magnetic field was applied to the sample so that both the in-plane and out-of-plane magnetic moments are aligned along a preferred direction. The magnetic field strengths were ± 4000 Oe along the out-of-plane, and ± 300 Oe along the in-plane direction. We obtained a total of 8 sets of data (4 magnetic field directions and 2 polarization setups), which were fitted using Supplementary eq. (7) and (8). Data and fits for the 7 nm–7 QL heterostructure sample are shown in Supplementary Fig. 4.

## Supplementary Note 4: Quantum well states and magnetic second harmonic generation

When the thickness of a material becomes finite (e.g. along the *z* direction), as in the case of the EuS-$Bi_2Se_3$ heterostructures, electrons can form a quantum well state (QWS) bound along the *z* direction. The energy dispersion along the *z* axis then becomes more discretised. As the thickness of the sample is varied, the number of discrete points in the band structure also changes, and the $k_z$ points are shifted accordingly (Supplementary Fig. 5). SHG is a two-photon process that becomes strongly enhanced near a resonant transition from one state in the occupied band to another state in the unoccupied band. Upon changing the thickness of the quantum well, optical transition takes place at different $k_z$ values, leading to a change in the SHG magnitude.

A more subtle effect on MSHG can be found in thin film heterostructures composed of both ferromagnetic and non-magnetic materials. In the past, large variations in the MSHG signal were measured in Co/Cu[5] and Au/Co/Au[6] thin film heterostructures across different sample thicknesses. In both cases, the MSHG intensity shows a strong oscillatory behavior as a function of the paramagnetic layer thickness. In the former case, the minority spin states of Co have a similar density of states (DOS) at the Fermi level as that of Cu, whereas the majority spin states have a very different DOS. Therefore, the quantum well confinement strengths of the two spin states are different from each other. As the thickness of the paramagnetic Cu layer is tuned, the DOS at the Fermi level of Cu correspondingly changes, and it in fact oscillates as a function of

thickness[7]. Therefore, the MSHG response in turn exhibits an oscillatory behavior as a function of the thickness of the paramagnetic layer in both the Co/Cu[5] and Au/Co/Au[6] heterostructures.

A similar argument can be applied to the fluctuating MSHG response from the EuS-$Bi_2Se_3$ heterostructures. Since EuS is a good electrical insulator, it is unlikely that a QWS resides inside the bulk of EuS, as most electrons are confined within the nuclei[8]. If the QWS forms within the $Bi_2Se_3$ film, the majority and minority spin states have different DOS's at the Fermi level when the $Bi_2Se_3$ film is magnetised through the proximity effect from the neighboring EuS film. The strength of the potential barrier exerted to each spin state is then different from each other. As the thickness of the EuS film is tuned, a change in the band structure of the EuS film can cause the relative confinement strength of the two spin states to become different, resulting in a strong change in the MSHG response.

## Supplementary References


1 McIver, J. W. *et al.* Theoretical and experimental study of second harmonic generation from the surface of the topological insulator $Bi_2Se_3$. *Phys. Rev. B* **86**, 035327 (2012).

2 Pan, R.-P., Wei, H. & Shen, Y. Optical second-harmonic generation from magnetized surfaces. *Phys. Rev. B* **39**, 1229-1234 (1989).

3 Pavlov, V., Pisarev, R., Kirilyuk, A. & Rasing, T. Observation of a Transversal Nonlinear Magneto-Optical Effect in Thin Magnetic Garnet Films. *Phys. Rev. Lett.* **78**, 2004-2007 (1997).

4 Hsieh, D. *et al.* Nonlinear Optical Probe of Tunable Surface Electrons on a Topological Insulator. *Phys. Rev. Lett.* **106**, 057401 (2011).

5 Wierenga, H. A. *et al.* Interface Magnetism and Possible Quantum Well Oscillations in Ultrathin Co/Cu Films Observed by Magnetization Induced Second Harmonic Generation. *Phys. Rev. Lett.* **74**, 1462-1465 (1995).

6 Kirilyuk, A., Rasing, T., Mégy, R. & Beauvillain, P. Nonlinear Magneto-Optical Response from Quantum Well States in Noble Metals: Double Period and Interface Localization. *Phys. Rev. Lett.* **77**, 4608-4611 (1996).

7 Bennemann, K.-H. *Non-linear optics in metals*. (Oxford University Press, 1998).

8 Mauger, A. & Godart, C. The magnetic, optical, and transport properties of representatives of a class of magnetic semiconductors: The europium chalcogenides. *Phys. Rep.* **141**, 51-176 (1986).
